# Optimization on production of konjac oligo‐glucomannan and their effect on the gut microbiota

**DOI:** 10.1002/fsn3.927

**Published:** 2019-01-25

**Authors:** Catarina Aprilia Ariestanti, Vatcharee Seechamnanturakit, Eni Harmayani, Santad Wichienchot

**Affiliations:** ^1^ Faculty of Agro‐Industry Prince of Songkla University Hat Yai, Songkhla Thailand; ^2^ Interdisciplinary Graduate School of Nutraceutical and Functional Food (IGS‐NFF) Prince of Songkla University Hat Yai, Songkhla Thailand; ^3^ Department of Food Science and Technology Faculty of Agricultural Technology Gadjah Mada University Yogyakarta Indonesia

**Keywords:** enzymatic hydrolysis, konjac oligo‐glucomannan, prebiotic index, short‐chain fatty acids

## Abstract

Konjac glucomannan (KGM) is a polysaccharide extracted from *Amorphophallus konjac,* and its degradation product is konjac oligo‐glucomannan (KOG). The aim of this study was to produce KOG from KGM and to evaluate its effect on the gut microbiota in fecal batch culture. KOG was produced by enzymatic hydrolysis using β‐mannanase. The optimum conditions were as follows: reaction temperature of 48°C, reaction time of 4 hr, pH of 5.5 and E/S of 0.05% followed by purification step using 3,000 NMWC ultrafiltration (UF) membrane pore size. The effect of KOG on changes in human fecal bacterial populations and short‐chain fatty acids (SCFAs) production was evaluated. The results showed that low‐molecular weight KOG (LKOG) from purification step with concentration of 9.54 mg/ml, and a prebiotic index (PI) of 0.76 was successfully produced. LKOG can enhance the production of butyric acid in the colon with the highest concentration (8.24 mM) found at 72 hr fermentation.

## 1 INTRODUCTION

Currently, sources of prebiotic can be found in most part of the world but not in Asia. Previous studies about prebiotics have concentrated on fructo‐oligosaccharides (FOS), galacto‐oligosaccharides (GOS), lactulose, inulin and some other potential prebiotics of carbohydrates (Connolly, Lovegrove, & Tuohy, 2010). Amongst these, glucomannans have gained attention due to its nontoxic and nonharmful properties. Glucomannans belong to the family of *Araceae* from genus of *Amorphophallus, Colocasia, Xanthosoma,* and *Alocasia* (Behera & Ray, 2016). Commercial glucomannan is extracted from dried tuber, particularly *Amorphophallus konjac* (Yanuriati, Wiseso, & Harmayani, 2017). Konjac glucomannan (KGM) is a polysaccharide derived from *A. konjac* which is mainly composed of mannose, and glucose is found in abundance in Asia particularly Southeast Asia (Tester & Al‐Ghazzewi, 2017). KGM has limited use due to its very high viscosity (Ojima et al., 2009). KGM has been used as emulsifier, thickener, or as nutritional supplements for constipation, high cholesterol and obesity patients (Behera & Ray, 2016). Human studies with glucomannan as dietary supplement have shown that glucomannan can promote weight loss in obesity patients and has the capability to reduce the risk of cancer and LDL cholesterol (Connolly et al., 2010) and selectively promote the growth of good bacteria in the gut so can act as prebiotic (Al‐Ghazzewi, Khanna, Tester, & Piggott, 2007).

Konjac oligo‐glucomannan (KOG) is the degradation product of KGM. Recent studies found that KOG with different molecular weights has different biological functions (Liu et al., 2015). Several methods have been developed to produce KOG by depolymerization of KGM such as acid degradation, oxidative degradation, physical methods, and enzymatic hydrolysis (Courtois, 2009; Liu, Xu, Zhang, Zhao, & Ding, 2016). Enzymatic hydrolysis has been the most widely used in the degradation of polysaccharides due to it can be safely produced oligosaccharides in room temperature (Liu et al., 2015). β‐mannanase randomly hydrolyze mannosidic linkages found in glucomannan (Jian, Zhu, Zhang, Sun, & Jiang, 2013). Response surface methodology (RSM) is a statistical protocol designed for experiments in which several factors can be varied concurrently. Box–Behnken design (BBD) is one of the experimental designs in analytical chemistry. BBD is efficient when large number of variables are involved (Bezerra, Santelli, Oliveira, Villar, & Escaleira, 2008). As a dietary fiber, KGM and KOG are indigestible in small intestine and then partially or completely fermented by colonic microflora; therefore, it can act as prebiotic (Mudgil & Barak, 2013). The ability of KOG to promote the growth of lactobacilli and bifidobacteria has been demonstrated previously (Al‐Ghazzewi et al., 2007). Al‐Sheraji et al. (2013) and Slavin (2013) showed that KOG has prebiotic capacity. The authors ascribed this prebiotic potential to its ability to stimulate the growth of probiotic bacteria thereby enhancing short‐chain fatty acids (SCFAs) production, reduce the number of pathogenic bacterial populations thereby improving the hosts immune system. Commercial KOG with health benefit is not available in the market due to its level of purity (Connolly et al., 2010). Ultrafiltration (UF) is one of the methods that has found wide application in separation and purification of polysaccharides due to the advantages it confers on the final product (Xing & Li, 2009).

The aim of this study was to find the optimum conditions necessary for the production of KOG using β‐mannanase and UF membrane, and then investigate its effect on the gut microbiota and in SCFAs.

## 2 MATERIALS AND METHODS

### 2.1 Materials

All chemicals and reagents were of analytical grade. About 95% purified konjac glucomannan was purchased from Pyson Co., Ltd., (Shaanxi, China). β‐mannanase was purchased from Mianyang Habio Bioengineering Co., Ltd., (Sichuan, China). Probes of DNA (Bif164, Lab158, Cris150, Bac303, and Eub338) used for fluorescent in situ hybridization (FISH) technique were purchased from Sigma‐Aldrich. Konjac glucomannan (P‐GLML) was purchased from Megazyme (Wicklow, Ireland) as standard. Porang glucomannan (PGM) as a comparation was a gift from Faculty of Agricultural Technology, Gadjah Mada University (Yogyakarta, Indonesia). Commercial inulin was purchased from BENEO‐Orafti (Belgium).

### 2.2 Production of konjac oligo‐glucomannan

Konjac glucomannan (1% w/v) was added into 0.2 M CH_3_COONa (sodium acetate) buffer and then mixed with β‐mannanase (E/S ranging from 0.025% to 0.1%, w/w) to start the reaction. The mixture was stirred continuously and incubated at pH 5.0–6.0 for 2–6 hr. The temperature of the water bath was maintained at 43–53°C. Enzyme was inactivated by boiling the samples for 10 min, and then, the samples were centrifuged at 8,000 *g* for 10 min. The supernatant was then analyzed using high‐performance size exclusion chromatography (HPSEC; Agilent model 1200 series, CA, USA) for KOG concentration and molecular weight distribution.

### 2.3 Experimental design

The hydrolysis parameters were optimized by RSM using four variables Box–Behnken factorial design (BBD) software version 16 (Minitab Pty Ltd, Sydney, Australia) to determine the optimum conditions for producing KOG. The parameters were temperature, time, pH, and E/S ratio.

### 2.4 Purification of konjac oligo‐glucomannan

Konjac oligo‐glucomannan was filtered using polysulfone (PSU) ultrafiltration (UF) hollow fiber membrane (GE Healthcare Pte Ltd., Singapore) with pore size of 10,000 and 3,000 NMWC (membrane effective area was 110 and 140 cm^2^, respectively). Ten thousand NMWC membrane was operated for 6 hr, and 3,000 NMWC membrane was operated for 9 hr. Trans‐membrane pressure (TMP) was set to 1.5 bar with feed tank temperature 50°C. Both retentate and permeate were collected and analyzed by HPSEC to determine the performance of the membrane in purification of KOG mixtures. Purified KOG was dried by spray dryer. Volume concentration factor (VCF) and rejection value (R_app_) were calculated according to the following equation (Millipore, 2003):

VCF=Total starting feed volume added to the operation/current retentate volume

$$R_{\mathrm{app}}=1-\left(\text{concentration in filtrate}/\text{concentration in feed}\right)$$

### 2.5 Preparation of fecal slurry

Fecal samples were collected from six healthy male and female donors who had not consumed probiotics, prebiotics or received antibiotics treatment for at least 3 months and had no digestive system disease. Fresh fecal samples were put into an anaerobic chamber and mixed with 0.1 M phosphate‐buffered saline (PBS; 8 g/L NaCl, 0.2 g/L KCl, 1.44 g/L Na_2_HPO_4_, and 0.24 g/L KH_2_PO_4_) pH 7.4 for the final concentration 10% (w/v). The slurry was homogenized by stomacher for 2 min and filtered with dialysis bag before inoculated into vessels.

### 2.6 In vitro fecal fermentation (batch culture) of konjac oligo‐glucomannan

Water jacket glass vessels were used and filled with basal medium contained per liter: 0.9 g peptone water, 0.9 g yeast extract, 0.045 g NaCl, 0.018 g K_2_HPO_4_, 0.018 g KH_2_PO_4_, 0.0045 g MgSO_4_.7H_2_O, 0.0045 g CaCl_2_.6H_2_O, 0.9 g NaHCO_3_, 0.225 g L‐cysteine. HCl, 0.225 g bile salts, 0.9 ml Tween 80, 4.5 μl vitamin K, 0.0225 g hemin, and 0.45 ml of 0.025% resazurin. The medium was dissolved in distilled water, adjusted to pH 7.0 and then sterilized by autoclave. Sterilized medium (90 ml) was placed and stirred magnetically into each of the vessels with temperature and pH controlled (37°C, pH 6.8) using circulated water bath and pH controller, respectively. The medium was maintained under an anaerobic environment by pumped in the nitrogen gas into vessel to imitate the conditions in colon (Connolly et al., 2010) overnight. The basal medium was inoculated with 10 ml of a 10% (w/v) fecal slurry. Substrates were added and dissolved in the basal medium with final concentration of 1% (w/v). The final volume of each culture was 100 ml. The sample (10 ml) from each vessel was taken at 0, 6, 12, 24, 48, and 72 hr for SCFAs analysis and enumeration of fecal bacteria by FISH technique. All samples were kept in −20°C.

### 2.7 Short‐chain fatty acids (SCFAs) analysis by high‐performance liquid chromatography (HPLC)

Samples from batch culture (1,125 μl) were centrifuged at 13,000 *g* for 15 min at 4°C. The supernatant was obtained and filtered through a 0.22 μm membrane nylon filter. Samples were analyzed for SCFA concentration for acetic, propionic, and butyric acid through HPLC system (Agilent model 1200 series, CA, USA) using an Aminex HPX‐87H ion exclusion column (Bio‐Rad, Richmond CA, USA) with a 7.8 mm diameter and 300 mm length at 50°C. Diode array detector (DAD) was used in the HPLC system with the UV absorbance was set at 215.4 nm. Mobile phase was 0.005 M H_2_SO_4_ with flow rate of 0.6 ml/min. The quantification of SCFAs of the samples were calculated from the calibration curves of acetic, propionic, and butyric acid.

### 2.8 Enumeration of fecal bacteria by fluorescent in situ hybridization (FISH) technique

Fluorescent in situ hybridization is a technique using 16S rRNA‐targeted oligonucleotide to evaluate the changes in different human fecal bacterial populations at six time points during fermentation of the substrates (Hugenholtz, Tyson, & Blackall, 2002). Fecal batch culture samples (375 μl) were mixed and fixed for 4–16 hr in cold 4% paraformaldehyde (pH 7.2) at 4°C in a ratio 1:3 of sample to 4% paraformaldehyde (v/v). The samples were then centrifuged at 13,000 *g* for 20 min at 4°C. Supernatant was removed, and the pellet was washed twice by resuspending in 1 ml phosphate‐buffered saline (PBS, pH 7.4) and centrifuging. The remaining pellet was resuspended in PBS/96% ethanol mix (1:1, v/v) and stored at −20°C for further use up to 3 months. The samples at the optimal dilution were mixed and dropped (20 μl) onto Teflon/Poly‐L‐Lysine coated six well microscope slides (Tekdon Inc., Myakka City, FL, USA). The samples on the slide were dried using slide warmer (Digicon MD‐700A, Thailand) at 46°C for 15 min and then dehydrated using alcohol series (50%, 80% and 96%) for 3 min in each concentration. Cells targeted with Lab158 probe was slightly modified by dropping 20 μl lysozyme into each well at room temperature for 15 min and the slides then washed in cool water (3 s) before dehydrated in alcohol series. After the evaporation of ethanol, the slides were returned to the slide warmer. Then 50 μl mixture of probe and hybridization buffer (1:9; v/v) were added to each well. Hybridization was performed in hybridization oven (Boekel Scientific InSlide Out Slide Hybridizer 241000, PA, USA) for 4 hr at appropriate temperature for the probes. The slides were washed with 50 ml of wash buffer for 15 min, then rinsed with cold water, and dried using compressed air. Antifade agent (10 μl; Invitrogen, USA) was applied to the surface of each well and covered with cover slip. The bacterial group was enumerated by fluorescence microscopy (Nikon E400 Eclipse, Nikon Instruments, Inc., New York, USA) and 15 randomized views from each well (Wichienchot, Prakobpran, Ngampanya, & Jaturapiree, 2017) were counted using NIS‐Elements BR 3.00, SP6 software (Nikon Instruments, Inc., New York, USA) for each sample.

Prebiotic candidate can be evaluated with the value of its prebiotic index (PI). Calculation of PI (Palframan, Gibson, & Rastall, 2003) is described below:

PI=(Bif/Total)+(Lac/Total)−(Bac/Total)−(Clos/Total)

where Bif is number of bifidobacteria*,* Lac is number of lactobacilli*,* Bac is number of bacteroides, and Clos is number of clostridia. The numerator is collected by the number of bacteria at sample time/number of bacteria at inoculation. Total bacteria are obtained by number of eubacteria count at sample time/number of eubacteria count at inoculation.

### 2.9 Statistical analysis

Statistical analysis was performed using the SPSS version 21.0 for windows (IBM Corp. New York, USA). The variation within the samples was analyzed using one‐way analysis of variance (ANOVA). Duncan's test was used to compare the means at 95% confidence level.

## 3 RESULTS AND DISCUSSION

### 3.1 Production of konjac oligo‐glucomannan

In this study, KOG was produced by enzymatic hydrolysis of KGM using β‐mannanase. The BBD experimental design was used to optimize the hydrolysis temperature, hydrolysis time, pH, and the initial enzyme to substrate ratio (E/S). The concentration of oligosaccharides after hydrolysis from each variation of the factors was analyzed by HPSEC, and the results are shown in Table 1. The highest oligosaccharides concentration was 9.21 mg/ml with the molecular weight of 1,552 Da ± 2.83 found at the condition of 48°C, pH of 5.5 and the E/S ratio of 0.05% (w/w) after 4 hr hydrolysis. The optimum conditions predicted by BBD for oligosaccharides and polysaccharides concentrations were 7.76 and 7.67 mg/ml, respectively. The predicted result was lower compared to one of the treatments described above, probably because the pH (6.0) and temperature (50.37°C) were higher than the optimum working conditions of the enzyme (pH 5.5, temperature 48°C).

**Table 1:** Concentration of oligosaccharides from enzymatic hydrolysis process

Treatment				
Time (hr)	Temp (°C)	pH	E/S (%)	Oligosaccharides concentration (mg/ml)
4	43	5.5	0.025	8.26
6	43	5.5	0.050	8.85
2	43	5.5	0.050	8.49
4	43	5.5	0.100	9.08
4	43	5.0	0.050	8.46
4	43	6.0	0.050	8.63
2	53	5.5	0.050	8.25
6	53	5.5	0.050	8.88
4	53	5.5	0.025	8.92
4	53	5.5	0.100	8.73
4	53	5.0	0.050	8.93
4	53	6.0	0.050	8.85
2	48	5.5	0.025	8.80
2	48	5.5	0.100	6.40
6	48	5.5	0.100	8.67
4	48	5.5	0.050	8.96
4	48	5.5	0.050	8.87
6	48	5.5	0.025	8.49
6	48	5.0	0.050	8.81
4	48	5.0	0.025	8.39
4	48	5.0	0.100	8.65
2	48	5.0	0.050	8.30
4	48	6.0	0.025	7.86
4	48	6.0	0.100	8.42
6	48	6.0	0.050	8.78
4	48	5.5	0.050	9.21
2	48	6.0	0.050	8.71

Chen et al. (2013) found that the increase of hydrolysis time, the activity of β‐mannanase decreased correspondingly. This is similar to our result. This study was successfully produced KOG by hydrolysis of KGM using β‐mannanase. Despite this method can be applied in the production of KOG, the product still contained unhydrolyzed KGM shown by the concentration of polysaccharides (data not shown). The results showed that β‐mannanase was able to hydrolyze KGM within 4 hr. The main reason for purifying KOG is to improve its market value and health benefits. Therefore, purification step is needed to purify KOG mixture to get the higher amount of oligosaccharide in the final product.

### 3.2 Purification of konjac oligo‐glucomannan

Konjac oligo‐glucomannan was purified using membrane technique based on the size exclusion mechanism. The purpose for this step was to purify the mixture of KOG by rejecting the polysaccharides in the product using the membrane pore size of 10,000 NMWC and 3,000 NMWC. As a result of the molecular weight of KGM (2.39 × 10^5 ^Da), polysaccharides and oligosaccharides were obtained in retentate and permeate, respectively. Both the retentate and permeate were analyzed by HPSEC to ascertain the level of purification obtained for KOG mixtures.

At the optimum KOG production (48°C, pH of 5.5, and E/S 0.05% for 4 hr), the initial product mixture contained 12.81 and 9.21 mg/ml of polysaccharides and oligosaccharides, respectively. The final concentration of polysaccharides and oligosaccharides during the purification step is shown in Table 2. The results indicate that the concentration of oligosaccharides in the permeate increased from 9.21 to 9.24 mg/ml with 10,000 NMWC membrane and from 9.21 to 9.54 mg/ml with 3,000 NMWC membrane. Additionally, the increased filtration time also improved the concentration of oligosaccharides in permeate. Volume concentration factor (VCF) of this study was 2.14 with the rejection value (R_app_) for 10,000 NMWC membrane pore size was −0.07 for oligosaccharides and 0.42 for polysaccharides. The R_app_ of oligosaccharides and polysaccharides from 3,000 NMWC membrane pore size was −0.12 and 0.58, respectively. The VCF represents the amount that the feed stream has been reduced in volume from the initial volume. Meanwhile, R_app_ represents the fraction of a particular substrate that is retained by the membrane. In order to obtain high yield of oligosaccharides, a low VCF and high R_app_ values are desirable (Millipore, 2003). Purification yield of oligosaccharides after UF step was 0.33% for 10,000 NMWC and 3.58% for 3,000 NMWC. In this study, the membrane with 10,000 NMWC pore size might not be effective to purify KOG because the concentration of oligosaccharides in the permeate was lower than the concentration obtained from the permeate of 3,000 NMWC. The results showed that the 3,000 NMWC membrane gave the highest concentration of KOG in the permeate; therefore, it was selected for the evaluation of prebiotic effect of KOG. A permeate with oligosaccharides concentration of 9.54 mg/ml contained oligosaccharides with low MW which later referred as low MW KOG (LKOG) according to MW inside the product (data not shown). Meanwhile, a retentate with oligosaccharides concentration of 8.49 mg/ml labeled as high MW KOG (HKOG) based on its MW (data not shown) that higher than LKOG. Jian et al. (2013) reported using the combination of γ‐irradiation and enzymatic hydrolysis were successfully produced KOG with MW lower than 2,200 Da. They found that 1,000 Da MWCO membrane could effectively separate and purify KOG. Our research produced KOG with MW of 1,552 Da ± 2.83 and the result was comparable with their study.

**Table 2:** Concentration of polysaccharides and oligosaccharides of KOG during purification

Concentration (mg/ml)		
Before UF	Polysaccharide	Oligosaccharide
	12.81	9.21

After UF	10,000 NMWC		3,000 NMWC	
	Polysaccharide	Oligosaccharide	Polysaccharide	Oligosaccharide
Permeate	7.93	9.24	5.65	9.54
Retentate	13.60	8.64	13.35	8.49

### 3.3 Prebiotic effect of konjac oligo‐glucomannan

Populations of fecal bacteria were counted in the fecal batch culture. The microbial groups counted were bifidobacteria, lactobacilli, bacteroides, clostridia, and eubacteria as total bacteria. Bifidobacteria and lactobacilli represent the beneficial bacteria of gastrointestinal microbiota. The pathogenic bacteria are represented by clostridia and bacteroides. The test substrates were LKOG, HKOG, KGM, PGM, and inulin as positive control. An additional vessel for negative control containing no carbon source was also tested. Prebiotic properties were evaluated by determination of the increase in number of beneficial bacteria and concentration of SCFAs during fermentation.

**Figure 1:**
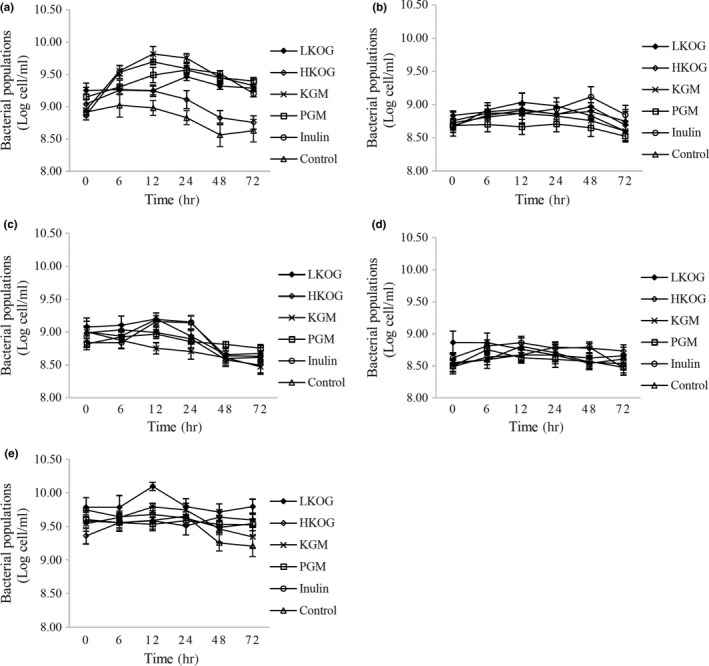
Fecal bacterial populations of (a) Bifidobacteria, (b) Lactobacilli, (c) Bacteroides, (d) Clostridia and (e) total bacteria in fecal batch culture fermentation

Figure 1a shows bacterial populations of bifidobacteria. Significant (*p *<* *0.05) growth was observed at 6 hr of fermentation for HKOG, KGM, PGM, and inulin and at 24 hr for LKOG. Populations of lactobacilli are shown in Figure 1b. The number of lactobacilli populations significantly increased at 48 hr for LKOG and at 6 hr for HKOG, KGM, and inulin. However, the increase of population was not significant for PGM. The bacteroides populations (Figure 1c) decreased significantly at 72 hr compared to 0 hr in all substrates fermentation. A significant decrease of clostridia populations (Figure 1d) was observed for both LKOG and inulin at 24 hr and at 12 hr for KGM and PGM. Total bacterial populations (Figure 1e) in LKOG were higher compared to other substrates at all time of fermentation. This indicated that LKOG could be fermented and used by gut microbiota to support their growth.

In 2018, the prebiotics market is approximated to reach $ 4.5 billion (Plongbunjong, Graidist, Knudsen, & Wichienchot, 2017). Prebiotics are functional food ingredients that modify the gut microbiota thus improve the host health (Sarbini & Rastall, 2011). Previous studies have investigated the health properties of KGM as a potential prebiotic with application in food and healthcare/pharmaceutical product (Behera & Ray, 2016; Jiang, Li, Shi, & Xu, 2018). Yang et al. (2017) showed KOG produced by enzymatic hydrolysis has the prebiotic effect by showing the ability to support the growth of lactobacilli and bifidobacteria. This result is comparable with ours. Recently Mao, Song, Yao, and Wu (2018) showed that KGM, and its degradation products almost have no influence to the growth of five strain of bifidobacteria. In this study, LKOG promoted the growth of bifidobacteria compared to their study, but its effect for the specific strain of bifidobacteria need further investigation. Previous study about prebiotic activity of PGM in vivo showed that PGM has prebiotic activity by suppressing the growth of *E. coli* (Harmayani, Aprilia, & Marsono, 2014). Compared to their study, our result showed the ability of PGM to promote the growth of bifidobacteria and significantly reduced the number of clostridia. In the present study, LKOG fermentation in the fecal batch culture could increase the number of beneficial bacteria and decrease pathogenic bacterial populations.

Prebiotic activities may have a contribution for health by promoting the growth of bifidobacteria and/or lactobacilli (Connolly et al., 2010). PI value can be used to evaluate the prebiotic activities by giving the quantitative score. Association between changes in beneficial bacteria with unwanted bacteria related to their beginning level was described as PI (Thitiratsakul & Anprung, 2014). Table 3 shows that PI of LKOG was 0.76. PI of KGM and PGM were higher than the hydrolyzed glucomannan (1.26 and 1.23, respectively). The PI of inulin as the common prebiotic was 0.97. The fecal batch culture showed that LKOG has prebiotic potency by showing the positive PI value and its fermentation increased the populations of beneficial bacteria. LKOG showed a prebiotic potential comparable with commercial inulin, KGM, and PGM.

**Table 3:** Prebiotic index scores of LKOG, HKOG, KGM, PGM, inulin, and control

Sample	Prebiotic index (PI)
LKOG	0.76
HKOG	0.47
KGM	1.26
PGM	1.23
Inulin	0.97
Control	0.41

### 3.4 Short‐chain fatty acids (SCFAs) production

Short‐chain fatty acids are the major products of bacterial fermentation from carbohydrates in the large intestine. Table 4a,b show the concentration of SCFAs in LKOG, KGM, inulin, HKOG, PGM, and control. Acetic acid is the highest SCFAs produced in the colon (Ríos‐Covián et al., 2016). In our study, acetic acid was the highest SCFAs produced in all substrates during fermentation. LKOG produced the highest acetic acid compared to other substrates. Propionic acid production required more specific substrate and bacterial group compared to acetic acid (Morrison & Preston, 2016). Propionic acid production increased significantly (*p *<* *0.05) at 6 hr for LKOG and at 12 hr for inulin. Butyric acid is the main source of energy for colonocytes (Al‐Sheraji et al., 2013). Butyric acid was produced after 6 hr from LKOG and increased significantly during the fermentation. The highest concentration of butyric acid from LKOG was 8.24 ± 0.12 mM at 72 hr, while inulin fermentation did not produce butyric acid. An increased of butyric acid in the gut has been linked to colon cancer and colonic inflammation reduction (Al‐Sheraji et al., 2013). The butyric acid produced from LKOG fermentation is higher than the butyric acid produced from the report of Connolly et al. (2010) probably because of the fermentation time from this study was longer and the MW of LKOG was lower than their study. The concentration of butyric acid from LKOG was higher than propionic acid. SCFAs production was in the order of acetic, butyric, and propionic acid. It is suggested that LKOG has different benefits with KGM and may have specific health function related to butyric acid production. Therefore, LKOG demonstrated prebiotic potential comparable to inulin.

**Table 4:** (a) Concentration of short‐chain fatty acids in batch culture fermentation of LKOG, KGM, and inulin. (b) Concentration of short‐chain fatty acids in batch culture fermentation of HKOG, PGM, and control

(a)									
Time (hr)	Concentration of SCFAs (mM)								
	Acetic acid			Propionic acid			Butyric acid		
	LKOG	KGM	Inulin	LKOG	KGM	Inulin	LKOG	KGM	Inulin
0	105.13 ± 0.03^a^	10.73 ± 1.28^b^	5.06 ± 0.05^d^	3.52 ± 0.04^b^	16.98 ± 0.93^a^	3.88 ± 0.17^c^	ND	ND	ND
6	100.89 ± 1.11^ab^	10.46 ± 0.37^b^	29.00 ± 0.34^c^	7.16 ± 1.93^a^	18.10 ± 0.32^a^	3.14 ± 0.16^c^	4.62 ± 1.62^b^	3.29 ± 0.18^a^	ND
12	95.23 ± 2.17^b^	42.90 ± 1.33^a^	34.83 ± 0.80^bc^	5.46 ± 0.11^ab^	5.36 ± 0.72^c^	7.44 ± 0.08^b^	5.52 ± 0.11^ab^	ND	ND
24	97.38 ± 2.29^b^	49.47 ± 0.30^a^	43.13 ± 3.56^a^	6.54 ± 0.78^ab^	6.94 ± 0.22^bc^	12.46 ± 1.40^a^	7.67 ± 1.02^a^	ND	ND
48	97.13 ± 2.34^b^	62.28 ± 0.26^a^	41.14 ± 1.10^ab^	6.36 ± 0.63^ab^	9.93 ± 1.34^b^	11.48 ± 0.19^a^	7.35 ± 0.13^ab^	ND	ND
72	105.76 ± 0.80^a^	44.30 ± 1.42^a^	39.15 ± 3.00^ab^	6.91 ± 0.51^a^	5.84 ± 2.36^bc^	10.24 ± 1.16^a^	8.24 ± 0.12^a^	ND	ND

(b)									
Time (hr)	Concentration of SCFAs (mM)								
	Acetic acid			Propionic acid			Butyric acid		
	HKOG	PGM	Control	HKOG	PGM	Control	HKOG	PGM	Control
0	43.74 ± 0.05^c^	12.28 ± 1.47^b^	3.03 ± 0.03^d^	3.70 ± 0.21^bc^	9.29 ± 0.12^a^	3.15 ± 0.02^a^	ND	3.65 ± 0.20^a^	ND
6	46.70 ± 0.83^c^	11.17 ± 0.00^b^	6.83 ± 0.28^c^	2.87 ± 0.01^c^	11.92 ± 0.50^a^	2.29 ± 0.12^c^	5.44 ± 0.37^b^	3.30 ± 0.06^b^	ND
12	50.76 ± 0.78^b^	28.37 ± 1.04^ab^	9.05 ± 0.11^b^	4.81 ± 0.06^ab^	2.99 ± 0.01^c^	2.52 ± 0.11^bc^	7.53 ± 0.20^a^	ND	ND
24	56.99 ± 1.61^a^	38.03 ± 1.13^a^	10.00 ± 0.58^b^	5.59 ± 0.14^a^	4.95 ± 0.09^c^	2.76 ± 0.01^ab^	2.89 ± 0.04^c^	ND	ND
48	53.69 ± 1.28^ab^	40.56 ± 0.42^a^	11.65 ± 0.48^a^	6.17 ± 0.71^a^	5.00 ± 0.18^c^	3.10 ± 0.23^a^	ND	ND	ND
72	55.98 ± 1.32^a^	51.75 ± 0.24^a^	12.72 ± 0.05^a^	6.29 ± 0.65^a^	6.37 ± 2.52^bc^	2.94 ± 0.00^a^	ND	ND	ND

*Notes*

Different letters of superscript in the same column mean value was significant difference (*p *<* *0.05).

ND: not detected.

## 4 CONCLUSIONS

Konjac oligo‐glucomannan was successfully produced by hydrolysis of KGM using β‐mannanase with the highest concentration of 9.54 mg/ml obtained under the conditions of 48°C, pH 5.5, hydrolysis time 4 hr, and E/S 0.05%. Low‐molecular weight KOG produced from UF membrane was selectively fermented by beneficial bacteria and can enhance the production of butyric acid during fecal fermentation. High concentration of KOG obtained under the optimum conditions showed the potential of this method to produce KOG for industrial scale. Further study of KOG to investigate its beneficial effect in human should be performed.

## CONFLICT OF INTEREST

The authors declare that they do not have any conflict of interest.

## ETHICAL REVIEW

This study does not involve any human or animal testing.
